# Imaging the Spatio-Temporal Dynamics of Supragranular Activity in the Rat Somatosensory Cortex in Response to Stimulation of the Paws

**DOI:** 10.1371/journal.pone.0040174

**Published:** 2012-07-19

**Authors:** M. L. Morales-Botello, J. Aguilar, G. Foffani

**Affiliations:** 1 Hospital Nacional de Parapléjicos, Servicio de Salud de Castilla-La Mancha, Toledo, Spain; 2 School of Biomedical Engineering, Science, and Health Systems, Drexel University, Philadelphia, Pennsylvania, United States of America; University of Salamanca- Institute for Neuroscience of Castille and Leon and Medical School, Spain

## Abstract

We employed voltage-sensitive dye (VSD) imaging to investigate the spatio-temporal dynamics of the responses of the supragranular somatosensory cortex to stimulation of the four paws in urethane-anesthetized rats. We obtained the following main results. (1) Stimulation of the contralateral forepaw evoked VSD responses with greater amplitude and smaller latency than stimulation of the contralateral hindpaw, and ipsilateral VSD responses had a lower amplitude and greater latency than contralateral responses. (2) While the contralateral stimulation initially activated only one focus, the ipsilateral stimulation initially activated two foci: one focus was typically medial to the focus activated by contralateral stimulation and was stereotaxically localized in the motor cortex; the other focus was typically posterior to the focus activated by contralateral stimulation and was stereotaxically localized in the somatosensory cortex. (3) Forepaw and hindpaw somatosensory stimuli activated large areas of the sensorimotor cortex, well beyond the forepaw and hindpaw somatosensory areas of classical somatotopic maps, and forepaw stimuli activated larger cortical areas with greater activation velocity than hindpaw stimuli. (4) Stimulation of the forepaw and hindpaw evoked different cortical activation dynamics: forepaw responses displayed a clear medial directionality, whereas hindpaw responses were much more uniform in all directions. In conclusion, this work offers a complete spatio-temporal map of the supragranular VSD cortical activation in response to stimulation of the paws, showing important somatotopic differences between contralateral and ipsilateral maps as well as differences in the spatio-temporal activation dynamics in response to forepaw and hindpaw stimuli.

## Introduction

To understand the basic elements of cortical somatosensory processing, it is necessary to study the spatio-temporal dynamics of cortical activation in response to somatosensory stimuli. Signals evoked by somatosensory stimuli can enter the cortex through several layers [1], [2], [3], but the main input is the granular layer (layer 4) [1], [4], [5]. From here somatosensory signals are distributed within cortical columns to supragranular layers (layers 2/3) [6], [7]. Supragranular layers then play a critical role in distributing the signals between cortical columns and to other regions involved in sensorimotor processing [8], [9], [10].

The spatio-temporal dynamics of supragranular cortical activation have been widely investigated *in vivo* using a relatively recent imaging technique: voltage-sensitive dye (VSD) imaging. VSD imaging allows the simultaneous imaging of the activation of large cortical regions with excellent spatial and temporal resolution [11], [12]. This resolution has made it possible to study in detail the spatio-temporal dynamics of spontaneous and evoked activation in the supragranular layers of the somatosensory cortex, especially in the barrel cortex [13]–[20]. More recently, VSD imaging has also been extended to the paw region of the primary somatosensory cortex to investigate cortical reorganization after stroke in mice [21], [22], [23] and after spinal cord injury in rats [24], [25], [26]. However, the exact spatio-temporal dynamics of supragranular cortical activation in response to contralateral and ipsilateral stimulation of the forepaw and hindpaw in physiological conditions remain unclear.

Two main issues are particularly relevant to fully understand both cortical reorganization after injury and sensorimotor integration in physiological conditions (e.g. during locomotion): (1) the comparison of contralateral vs ipsilateral responses, and (2) the comparison of responses to forepaw vs hindpaw stimuli. On the one hand, ipsilateral responses could originate below the level of the thalamus [27], [28], at thalamocortical level, or at cortical level from projections through the corpus callosum [29]–[32]. The presence of multiple possible pathways by which somatosensory inputs could reach the ipsilateral cortex suggest that the cortical map of the ipsilateral body might not be perfectly symmetrical to the cortical map of the contralateral body [33]. On the other hand, two main anatomical differences distinguish the forepaw and the hindpaw regions of the rat primary somatosensory cortex. First, the forepaw region is larger than the hindpaw region [34]. Second, the forepaw somatosensory cortex is mostly separated from the corresponding region of the motor cortex, whereas most of the hindpaw somatosensory cortex overlaps with the corresponding region of the motor cortex [35], [36]. Because of the known projections from the somatosensory cortex to the motor cortex [17], [37]–[41], it therefore seems reasonable to hypothesize that the spatio-temporal dynamics of supragranular cortical activation in response to stimulation of the forepaw compared to stimulation of the hindpaw will be different.

In the present work we employed VSD imaging to investigate in detail the spatio-temporal dynamics of supragranular cortical activation in response to stimulation of the paws in normal urethane-anesthetized rats. Specifically, our main points of interest were: (1) to determine the supragranular VSD response amplitudes and latencies to stimulation of the four paws, (2) to compare the spatial representation of contralateral vs. ipsilateral VSD responses, (3) to assess the extent of cortical activation and the amount of overlap between the forepaw and hindpaw regions, and (4) to compare the directionality in the cortical activation dynamics between forepaw and hindpaw stimuli. This study provides a complete description of the spatio-temporal dynamics of VSD activation of the rat supragranular somatosensory cortex in response to stimulation of the paws.

## Results

### VSD Imaging of Cortical Somatosensory Responses

**Figure 1:**
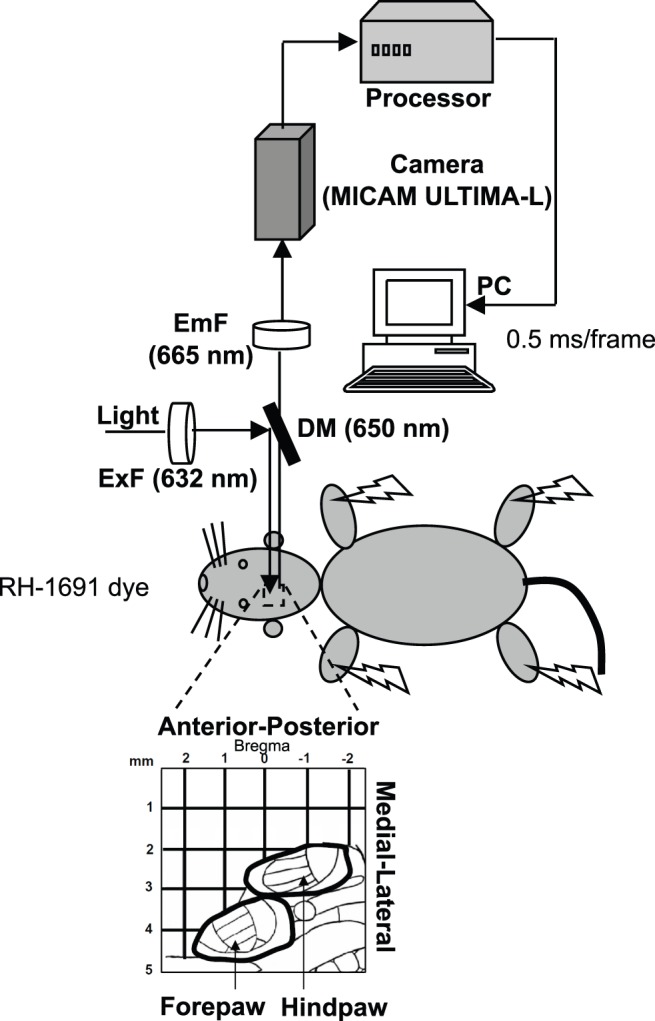
Experimental design. We imaged the VSD signals from the somatosensory cortex of one hemisphere of the rat in response to electrical stimulation of the paws. To image the VSD signals, the light was band-pass filtered at 632 nm by an excitation filter (ExF) and reflected toward the cortex by a 650 nm dichroic mirror (DM). The emitted fluorescence was transmitted through the dichroic mirror, subsequently long-pass filtered at 665 nm by an emission filter (EmF), and finally imaged using a MICAM ULTIMA-L system composed by a high-speed camera and a corresponding processor unit (BrainVision Inc.). A 5-mm×5-mm cortical area was imaged, as schematically represented at the bottom. The black lines delimit the forepaw and hindpaw regions in the somatotopic map of the rat primary somatosensory cortex.

**Figure 2:**
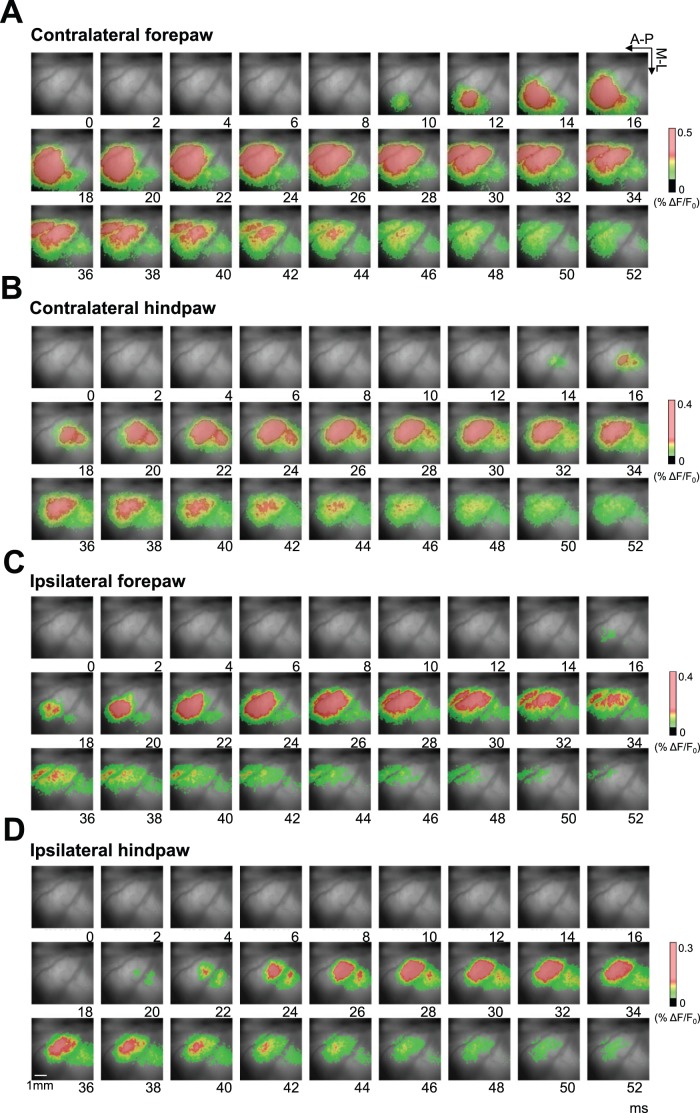
VSD imaging of cortical somatosensory responses. Cortical activation dynamics evoked by high intensity stimulation of the contralateral forepaw (A), contralateral hindpaw (B), ipsilateral forepaw (C) and ipsilateral hindpaw (D) in the first 50 ms after the stimuli (0 represent the stimulus onset) in a representative animal. The arrows indicate the anterior-posterior and medial-lateral directions. Each image corresponds to the average of 30 trials. The initial activation evoked by stimulation of the contralateral forepaw and hindpaw corresponds to the somatotopic map of the rat primary somatosensory cortex, however, we found a different somatotopic map in the activation evoked by stimulation of the ipsilateral paws. In few milliseconds the activation expanded reaching large cortical regions, with a different directionality between forepaw and hindpaw cortex.

The VSD signal is proportional to the membrane potential changes and to the membrane area of the neuronal elements stained under each measured pixel [12], [42]. In vivo, this signal primarily represents the activity of the supragranular layers 2/3 [13], [43], [44] and, more specifically, the dendritic activity of pyramidal cells because they provide the greatest contribution to the imaged membrane area [11], [12], [13]. However, VSD is not sensitive to spikes, as they make up a small percentage of the total change of the measured membrane potential [13], [45]. Rather, VSD signals are similar (proportional) to the local field potential recorded electrophysiologically [12], [13], [18], [43], [45], [46]. Both represent population activity, and both methods can measure with good temporal resolution (VSD imaging reaches 0.1 ms). However, VSD imaging also makes it possible to simultaneously image a wide cortical region with great spatial resolution (>50 µm) [12]. This would be equivalent to placing 10,000 electrodes in a 5-mm×5-mm area. Here we employed VSD imaging to record the activation of the rat supragranular somatosensory cortex in response to electrical stimuli at high intensity (6 mA) and low intensity (0.6 mA) separately delivered to the four paws (Fig. 1). We will first provide a qualitative description of the cortical activation dynamics using a representative example (Fig. 2).

Stimulation of the contralateral forepaw (Fig. 2A) initially activated a region that was more anterior and lateral compared to the region initially activated by stimulation of the contralateral hindpaw (Fig. 2B). The separation between these initially activated regions, which we define as foci, was approximately 2 mm. This is consistent with the known somatotopic organization of the rat primary somatosensory cortex. Within a few milliseconds, the activation expanded and reached beyond the somatosensory cortex to a large extent. This expansion was not uniform, but it occurred in two principal directions: most importantly in the medial direction but also in the posterior direction. The non-uniform directionality was more clearly observable in response to forepaw stimuli than in response to hindpaw stimuli.

Unlike contralateral stimulation, ipsilateral stimulation initially activated two foci that were well separated and did not match with the contralateral stimulation focus (Fig. 2C,D). One of the ipsilateral foci was medial to the contralateral focus and was localized within the motor cortex. The other ipsilateral focus was posterior and was localized within the somatosensory cortex. Within few milliseconds, the activations from these foci extended, joining together and reaching a large cortical region, although the overall extension was smaller compared to contralateral stimulation.

In the following sections, we will first show the quantitative results concerning the response measures that are typically employed in electrophysiological studies, such as response amplitudes and response latencies. Then, we will show the quantitative results regarding the spatio-temporal aspects of the cortical activation, which are possible to measure using VSD imaging.

### Amplitudes and Latencies of Cortical Somatosensory VSD Responses

**Figure 3:**
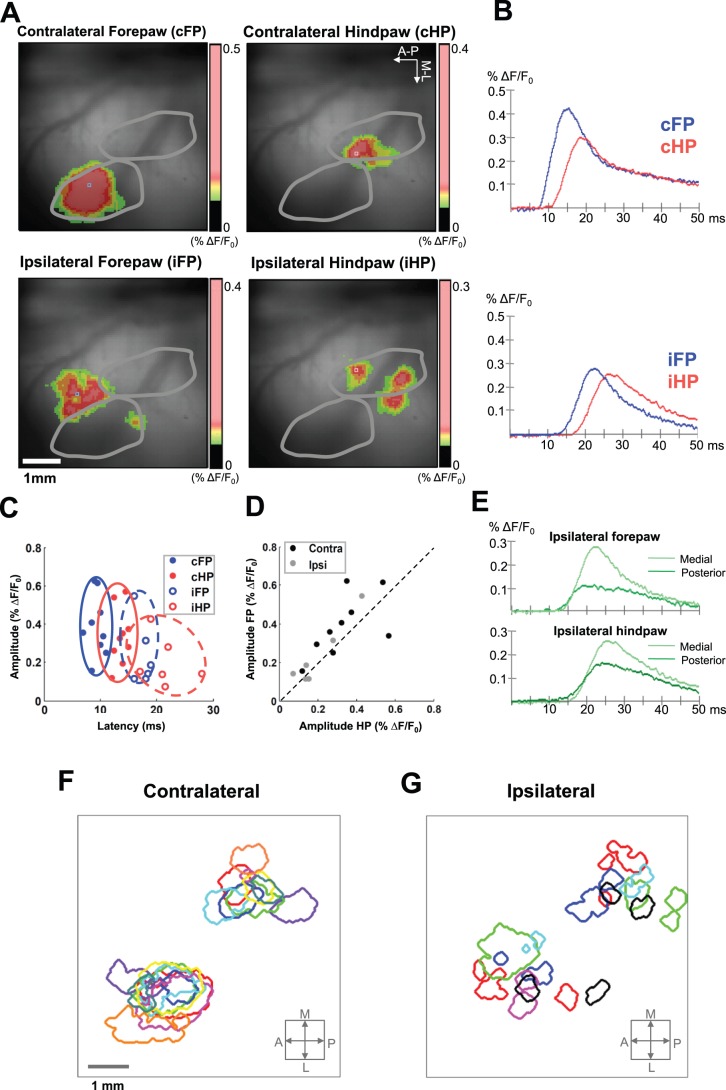
Amplitudes and latencies of cortical somatosensory VSD responses. (A) Cortical activation evoked by high-intensity stimulation of the paws in the first 2–3 ms after activation onset in a representative animal. The gray lines delimit the forepaw and hindpaw regions in the somatotopic map of the rat primary somatosensory cortex. The arrows indicate the anterior-posterior and medial-lateral directions. Ipsilateral stimuli activated two separate regions, one more medial and the other more posterior than the region activated by contralateral stimuli. (B) Temporal evolution of the responses in the corresponding points of maximal activation intensity. (C) Amplitude versus latency of the responses to stimulation of contralateral and ipsilateral paws represented for all rats. (D) Amplitude of the response to stimulation of the contralateral and ipsilateral forepaw with respect to the amplitude of the response to stimulation of contralateral and ipsilateral hindpaw, represented for all rats. Forepaw stimuli evoked responses with bigger amplitude and shorter latency compared with hindpaw stimuli (for both contralateral and ipsilateral stimuli). (E) Temporal evolution of the response in the point with maximal activation intensity in each of the two foci (medial and posterior to contralateral focus) evoked by stimulation of ipsilateral forepaw and hindpaw in a representative animal. The medial focus displayed larger amplitude but longer latency than the posterior focus. In all time axes, zero indicates stimulus onset. (F,G) Initial activation maps for high-intensity contralateral stimuli (F) and ipsilateral stimuli (G) from all animals. Antero-lateral activations (lower left) correspond to forepaw stimulation, postero-medial activations (upper right) correspond to hindpaw stimulation. Each color is a different animal. Because we did not have an external stereotaxic reference, for illustration purposes the initial activations (1 ms) were realigned so that for each activation the center of the image corresponds to focus activated by high-intensity stimulation of the opposite contralateral paw (i.e. the center of the image is the contralateral hindpaw focus for the initial activations evoked by both contralateral and ipsilateral forepaw stimuli, and it is the contralateral forepaw focus for the initial activation of both contralateral and ipsilateral hindpaw stimuli).

We applied electrical stimuli to the rats’ paws, and we measured the amplitude, initial latency and peak latency of the responses (see Material and methods). Fig. 3A shows an example of cortical activation by high intensity stimulation of the contralateral and ipsilateral paws –2–3 ms after the initial activation – and Fig. 3B shows the temporal evolution of the VSD responses in the points of maximal intensity of the foci. The amplitude, initial latency and peak latency in response to high- and low-intensity stimuli are reported in Table 1.

**Table 1:** Amplitudes and latencies of cortical VSD responses to electrical stimulation of the paws.

	HIGH INTENSITY (6 mA)			LOW INTENSITY (0.6 mA)		
	Amplitude (% DF/F)	Latency (ms)	Peak latency (ms)	Amplitude (% DF/F)	Latency (ms)	Peak latency (ms)
**cFP**	0.39±0.16	9.40±0.65	16.67±2.98	0.28±0.15	16.20±7.21	27.56±7.83
**cHP**	0.33±0.15	13.50±1.22	20.06±1.88	0.19±0.11	23.20±5.13	33.61±11.33
**iFP**	0.24±0.17	17.58±1.28	24.50±2.30	-	-	-
**iHP**	0.20±0.13	21.42±3.72	30.67±4.40	-	-	-

(cFP = contralateral forepaw, cHP = contralateral hindpaw, iFP = ipsilateral forepaw, iHP = ipsilateral hindpaw).

The VSD response amplitude to contralateral forepaw stimuli was significantly larger than to contralateral hindpaw stimuli (two-way ANOVA, forepaw-hindpaw factor: *p* = 0.0430, n = 9) (Fig. 3C,D) and was significantly larger with high-intensity than with low-intensity stimuli (two-way ANOVA, high intensity-low intensity factor: *p* = 0.0308, n = 9). The VSD response latency was significantly shorter for contralateral forepaw stimuli than for contralateral hindpaw stimuli (two-way ANOVA, forepaw-hindpaw factor; initial latency: *p*<0.0001, n = 9; peak latency: *p* = 0.0004, n = 9) (Fig. 3C) and was shorter with high-intensity than with low-intensity stimuli (two-way ANOVA, high intensity-low intensity factor; initial latency: *p*<0.0001, n = 9; peak latency: *p* = 0.0002, n = 9).

To consistently compare the responses to ipsilateral and contralateral stimulation, we also selected the focus with maximal signal value in response to ipsilateral stimulation, and we determined the response amplitude and response latency. In comparison with contralateral stimuli, the response amplitudes evoked by ipsilateral stimuli were significantly smaller (two-way ANOVA, contralateral (n = 9)-ipsilateral (n = 6) factor: *p* = 0.0082), and the latencies were significantly larger (two-way ANOVA, contralateral (n = 9)-ipsilateral (n = 6) factor: *p*<0.0001 for both initial and peak latency) (Fig. 3C,D).

To investigate possible differences between the responses in the two foci evoked by ipsilateral stimulation, we selected the point with maximal signal value in each of the foci (medial and posterior), and we studied the response amplitude and response latency of these points. We found that the response amplitude was slightly larger in the medial (motor) than in the posterior (somatosensory) focus (two-way ANOVA, medial focus-posterior focus factor: *p* = 0.0334, n = 6; forepaw-hindpaw factor: *p* = 1.00, n = 9; interaction: p = 0.077), but interestingly, the half-peak response latency in the posterior (somatosensory) focus was smaller than in the medial (motor) focus (two-way ANOVA, medial focus-posterior focus factor: *p* = 0.0040, n = 6; forepaw-hindpaw factor: *p* = 0.0118, n = 9; interaction: p = 0.63) (Fig. 3E). The amplitude and latencies of the responses to ipsilateral stimuli, separating the two foci, are reported in Table 2. Initial contralateral and ipsilateral activation maps from all the animals are reported in Fig. 3F,G.

**Table 2:** Amplitudes and latencies in the two foci of ipsilateral responses.

	IPSILATERAL FOREPAW		IPSILATERAL HINDPAW	
	Amplitude (% DF/F)	Half peak latency (ms)	Amplitude (% DF/F)	Half peak latency (ms)
**Medial (motor) focus**	0.22±0.16	19.83±1.81	0.20±0.13	23.75±2.91
**Posterior (somatosensory) focus**	0.16±0.14	18.75±1.84	0.19±0.14	22.00±2.05

### Extent of Cortical Somatosensory VSD Responses

The somatosensory stimuli initially evoked a localized activation that later spread to larger cortical regions beyond the somatosensory cortex.

**Figure 4:**
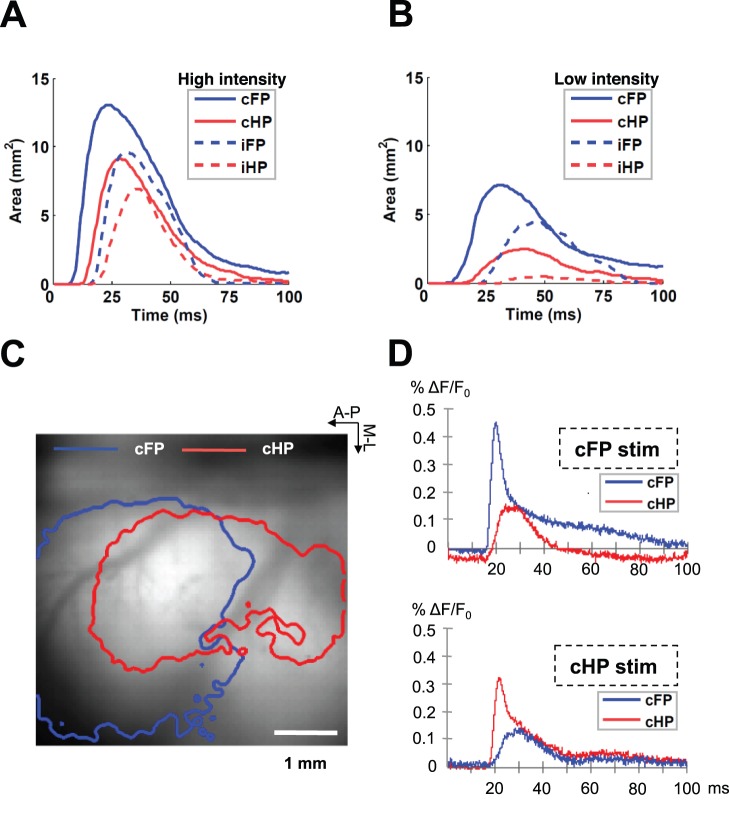
Extent of cortical somatosensory VSD responses. (A,B) Temporal evolution of the activated area in the first 100 ms of the response to stimulation of contralateral forepaw (cFP), contralateral hindpaw (cHP), ipsilateral forepaw (iFP) and ipsilateral hindpaw (iHP) at high-intensity (A) and low-intensity (B). The curves are the average of all animals. The extent of the activation was greater and faster with forepaw compared to hindpaw stimulation and with contralateral compared to ipsilateral stimulation. (C) Cortical overlap between the maximal activated regions by stimulation of contralateral forepaw and hindpaw in a representative animal. The arrows indicate the anterior-posterior and medial-lateral directions. (D) Temporal evolution of the responses in the forepaw focus and in the hindpaw focus evoked by stimulation of both contralateral forepaw (left) and hindpaw (right) in a representative animal. The stimulation of a paw evoked responses not only in the corresponding cortical region but also in the cortical region corresponding to the other paw.

First, we calculated the area of the cortical region activated by stimulation of the contralateral forepaw and hindpaw during the first one hundred milliseconds of the response (Fig. 4A,B). The maximal activated area due to forepaw stimulation was larger than that due to hindpaw stimulation (two-way ANOVA, forepaw-hindpaw factor: *p* = 0.0084, n = 9). The maximal activated area using a high-intensity stimulus was larger than when using a low-intensity stimulus (two-way ANOVA, high intensity-low intensity factor: *p* = 0.0006, n = 9). The corresponding values of the maximal activated areas and the latency at which the maximal area was reached are reported in Table 3. Similar differences were observed for the maximal activated areas due to ipsilateral stimuli, which were overall smaller compared to contralateral stimuli (Fig. 4A,B).

**Table 3:** Spatio-temporal measures of the extent of cortical somatosensory VSD responses to contralateral stimuli.

		HIGH INTENSITY	LOW INTENSITY
**Area _MAX_ (mm^2^)**	**FP**	14.38±4.91	9.04±6.83
	**HP**	9.67±4.64	3.02±3.23
**Latency_MAX_ (ms)**	**FP**	23.89±5.44	34.94±9.25
	**HP**	27.67±4.60	41.00±7.71
**Activation velocity_MAX_ (mm^2^/ms)**	**FP**	2.44±0.66	1.16±1.00
	**HP**	1.30±0.49	0.34±0.29
**Maximal cortical overlap (mm^2^)**	**FP-HP**	7.51±4.44	0.52±0.57

(FP = forepaw, HP = hindpaw).

The greater slope in the curve corresponding to the temporal evolution of the activated area in response to forepaw stimuli suggested a faster activation compared to hindpaw stimuli (Fig. 4A,B). To quantify the activation velocity, we calculated the derivative of the area with respect to time. The maximal activation velocity for contralateral forepaw stimulation was significantly larger than for hindpaw stimulation (two-way ANOVA, forepaw-hindpaw factor: *p* = 0.0017, n = 9) and was significantly larger using high-intensity compared with low-intensity stimulation (two-way ANOVA, high intensity-low intensity factor: *p*<0.0001, n = 9). The values of the maximal activation velocity are shown in Table 3.

The great extent of cortical activations in response to somatosensory stimuli suggested that a large overlap exists between the supragranular cortical regions activated by stimulation of the different paws. We quantified the overlap between the maximal activated areas due to contralateral forepaw and hindpaw stimulation (Fig. 4C). The maximal cortical overlap was larger with high-intensity than with low-intensity stimulation (*t*-test: *p* = 0.0317, n = 9). The values of maximal cortical overlap are reported in Table 3.

With high-intensity stimuli, the cortical activation evoked by contralateral forepaw stimulation reached the hindpaw focus in 100% of the animals, and the cortical activation evoked by hindpaw stimulation reached the forepaw focus in 44% of the animals (Fig. 4D). A stimulus to the forepaw evoked a VSD response in the hindpaw focus with an amplitude that was 61% smaller (*t*-test: *p*<0.0001, n = 9) and 8.61±2.88 ms slower (*t*-test: *p*<0.0001, n = 9) than in the forepaw focus, and a stimulus to the hindpaw evoked a VSD response in the forepaw focus with an amplitude that was 67% smaller (*t*-test: *p* = 0.0042, n = 4) and 11.13±4.80 ms slower (*t*-test: *p* = 0.0037, n = 4) than in the hindpaw focus. Taking into account the distance between the forepaw focus and hindpaw focus (2.15±0.50 mm), we determined that the linear activation velocity from the forepaw focus to the hindpaw focus (0.12±0.04 mm/ms, n = 9) was larger than the linear activation velocity from the hindpaw focus to the forepaw focus (0.08±0.01 mm/ms, n = 4) (*t*-test: *p* = 0.0112).

### Directionality of Cortical Somatosensory VSD Responses

**Figure 5:**
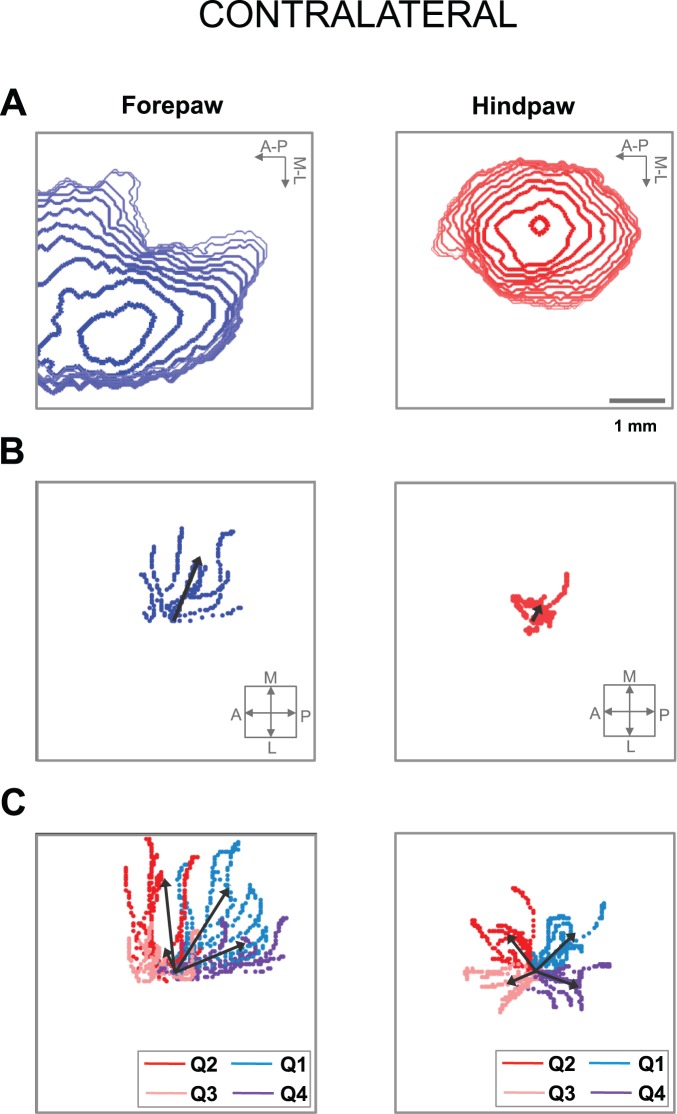
Directionality of cortical somatosensory VSD responses to contralateral stimuli. (A) Contour maps of the temporal evolution of the region activated by stimulation of the contralateral forepaw (left) and hindpaw (right) in a representative animal. The contours are displayed each 1 ms from the initial activation until the activated area reached the maximal value. The arrows indicate the anterior-posterior and medial-lateral directions. (B) Spatio-temporal evolution of the global activation center for forepaw (left) and hindpaw (right) stimuli until the activated area was maximal, in all animals. (C) Spatio-temporal evolution of the activation center in the four quadrants (Q1–Q4) for forepaw (left) and hindpaw (right) stimuli until the activated area was maximal, in all rats. In B,C the initial activation centers corresponding to each rat were aligned and placed in the center of the image and the arrows into the small square indicate the anterior-posterior and medial-lateral directions.

The somatosensory stimuli evoked cortical activations that spread non-uniformly through the cortex. Here, we studied the possible directionality of the activation dynamics, focusing on the response to contralateral stimulation. We first constructed contour maps of the temporal evolution of the activated region (see Materials and methods; Fig. 5A). We clearly observed a different directionality in the activation dynamics between forepaw and hindpaw stimulation.

To quantitatively study this directionality, we calculated the global activation center in each instant and followed its spatio-temporal evolution until the activated area reached the maximal value (see Material and methods; Fig. 5B; Table 4). The activation dynamics for forepaw stimulation involved a significant movement of the global activation center in the medial direction (*t*-test: medial-lateral, *p* = 0.0001, n = 9), and a tendency of smaller movement in the posterior direction (*t*-test: anterior-posterior, *p* = 0.06, n = 9). Conversely, there was no significant movement of the global activation center for hindpaw stimulation in either direction (*t*-test: medial-lateral, *p* = 0.12, n = 9; anterior-posterior, *p* = 0.38, n = 9).

**Figure 6:**
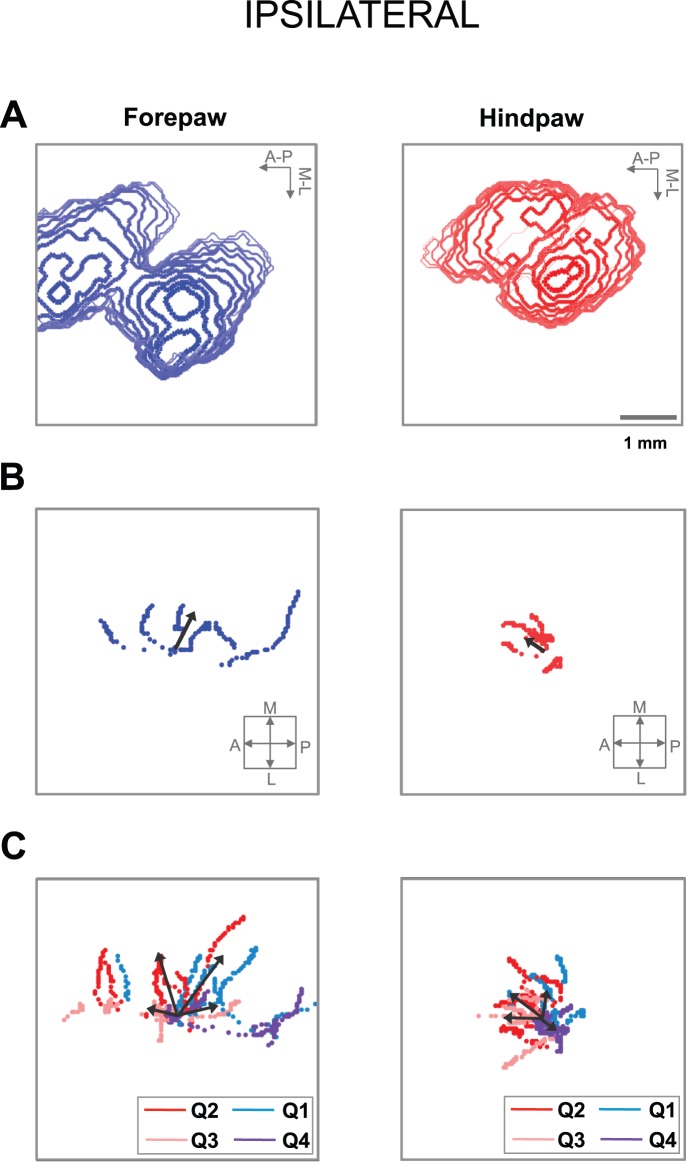
Directionality of cortical somatosensory VSD responses to ipsilateral stimuli. (A) Contour maps of the temporal evolution of the region activated by stimulation of the ipsilateral forepaw (left) and hindpaw (right) in a representative animal. (B) Spatio-temporal evolution of the global activation center for forepaw (left) and hindpaw (right) stimuli until the activated area was maximal, in all animals. (C) Spatio-temporal evolution of the activation center in the four quadrants (Q1–Q4) for forepaw (left) and hindpaw (right) stimuli until the activated area was maximal, in all rats.

As the spread was not uniform, the global activation center did not necessarily yield a complete description of the activation direction. Therefore, we followed the spatio-temporal evolution of the activation center in each of the four quadrants defined by the anterior-posterior and medial-lateral axes around the global activation center (see Material and methods; Fig. 5C; Table 4). Consistent with the results obtained in the previous paragraph, the spread of activation due to forepaw stimulation had a net evolution in the medial direction, whereas the spread of activation due to hindpaw stimulation was more homogeneous in all directions. different directionality in the activation dynamics between forepaw and hindpaw stimulation. Similar differences of activation dynamics between forepaw and hindpaw stimulation were observed when we analyzed the responses to ipsilateral stimulation (Fig. 6).

**Table 4:** Directionality of cortical somatosensoy VSD responses to contralateral stimuli.

	MEDIAL-LATERAL		ANTERIOR-POSTERIOR	
	Forepaw	Hindpaw	Forepaw	Hindpaw
**Global activation center (mm)**	1.17±0.47	0.23±0.35	−0.46±0.67	−0.10±0.30
**Activation center, Q1 (mm)**	1.89±0.69	0.80±0.43	−1.45±0.66	−0.88±0.34
**Activation center, Q2 (mm)**	2.04±0.59	0.78±0.42	0.48±0.64	0.63±0.39
**Activation center, Q3 (mm)**	0.37±0.29	−0.38±0.31	0.45±0.69	0.72±0.46
**Activation center, Q4 (mm)**	0.39±0.33	−0.38±0.32	−1.55±0.72	−0.94±0.29

(the values represent the maximal movement of the global activation center and of the activation center in the four quadrants with respect to the initial global activation center. Q1–Q4 corresponds to quadrants 1 to 4 and positive values indicate medial or anterior movement and negative values indicate lateral or posterior movement).

## Discussion

The aim of this study was to determine the spatio-temporal activation dynamics of the supragranular somatosensory cortex in response to stimulation of the paws in anesthetized rats using VSD imaging. Our main results suggest differences between the somatotopic cortical maps corresponding to the contralateral and ipsilateral paws and a different directionality in the activation dynamics in response to forepaw and hindpaw stimuli. These findings offer a complete spatio-temporal map of the VSD activation of the supragranular somatosensory cortex after stimulation of the paws in physiological conditions.

### Amplitudes and Latencies of Cortical Somatosensory VSD Responses

Stimulation of the contralateral forepaw evoked VSD responses with greater amplitude and smaller latency than stimulation of the contralateral hindpaw. The latency difference is consistent with previous observations in the thalamus [47] and in the infragranular cortex [48] and is likely due to the greater distance from the hindpaw to the brain stem compared with the forepaw. The amplitude difference could be explained by the higher density of peripheral innervation of the forepaw compared to the hindpaw, which may correspond to a more extensive arborization through the superior levels [49]. In fact, at the cortical level, there is a more dense packing of pyramidal cells in the representation of the forepaw compared with the hindpaw [50]. Electrophysiological studies of the infragranular layers have similarly shown larger responses to forepaw stimulation compared with hindpaw stimulation both with single-neuron recordings [48] and with local field potentials [51]. Moreover, studies employing intrinsic optical imaging [52] and BOLD fMRI [53] have also shown the same result. Larger responses to forepaw compared to hindpaw stimuli could therefore represent a general property of the rat somatosensory system.

The responses to the stimulation of ipsilateral paws differed compared with the responses to contralateral stimulation in two principal ways. First, the response to ipsilateral stimulation had a smaller amplitude and greater latency than the response to contralateral stimulation, as expected based on previous electrophysiological studies in the infragranular cortex [48], [54]. Second (and more interesting), while the contralateral stimulation initially activated only one focus, the ipsilateral stimulation activated two foci. One focus, medial to the focus activated by contralateral stimulation, was stereotaxically localized in the motor cortex. Interestingly, this focus was activated with a greater amplitude but with a slightly longer latency than the other focus, which was posterior to the focus activated by contralateral stimulation and stereotaxically localized in the somatosensory cortex.

Even though we cannot exclude subtle differences in electrode placement between the two sides of the body, it is improbable that they could contribute to the differences between ipsilateral and contralateral responses, because our high-intensity stimuli likely activated almost all fibers ascending through the paws. Furthermore, possible differences in electrode placement cannot justify the presence of two foci in the ipsilateral responses. Spatial differences between ipsilateral and contralateral activations are therefore likely to be due to innervations asymmetries. Indeed, the low level of overlap between the foci activated by contralateral and ipsilateral stimulation is consistent with the low colocalization of responses in the infragranular layers to tactile stimulation of contralateral and ipsilateral forepaw locations [33].

The activation of the somatosensory cortex by stimulation of the contralateral and ipsilateral paws has already been studied using VSD imaging by Ghosh et al. [24] in isoflurane-anesthezed rats. However, that previous work principally reports activation of the motor cortex in response to ipsilateral stimulation – which is indeed consistent with our findings – without observing two foci. It seems unlikely that Ghosh et al. did not report two foci due to the different anesthesia. A more probable explanation could be the lower temporal resolution employed in their study (5 ms) compared to ours (0.5 ms), because of two main reasons: (a) it did not allow for the more subtle spatio-temporal aspects of the cortical activations to be observed and (b) it required them to use much lower stimulus strengths compared to our high-intensity stimuli to avoid saturation of the VSD signals. Overall, the multiple foci of ipsilateral activation reported here complete the picture provided by Ghosh et al. and could be explained by the multiple origins, both cortical [55], [32], [56] and subcortical [57]–[59], [28], of ipsilateral responses.

Thus, we conclude that there are important differences in the supragranular somatotopic maps that represent the contralateral and ipsilateral paws. In the following sections we will focus on the possible differences in spatial-temporal activation dynamics in response to stimulation of the forepaw and hindpaw.

### Extent of Cortical Somatosensory VSD Responses

Forepaw and hindpaw somatosensory stimuli activated large areas of the sensorimotor cortex, well beyond the forepaw and hindpaw somatosensory areas of classical somatotopic maps [34]. From a mechanistic perspective, these extensive activations can be understood by the fact that VSD signals reflect subthreshold activity in the supragranular layers [13]. In these layers, the pyramidal neurons have broad subthreshold receptive fields [60] and long-range horizontal connections [10], [61], [62].

These extensive activations imply a large overlap between cortical areas activated by forepaw and hindpaw stimuli, consistent with previous electrophysiological observations in the infragranular cortex [48]. Indeed, in our experiments stimulation of the forepaw evoked a supragranular activation of a larger area than that evoked by stimulation of the hindpaw. This result is consistent with the largest representation of the forepaw throughout the somatosensory system [49], [63], [64], and with recent results obtained with fMRI [53]. Extensive activations and overlaps seen at the level of the entire sensorimotor cortex are directly related to the large receptive fields seen at the single neuron level [48], [33], which have been suggested to be important for constructing simple yet sophisticated spatio-temporal codes for somatosensory processing [65], [66]. The cortical overlap observed here could also be relevant for forepaw-hindpaw sensorimotor integration.

Not only forepaw stimuli activated larger cortical areas than hindpaw stimuli, but also the activation velocity was greater for forepaw stimuli than for hindpaw stimuli. Consequently, the linear activation velocity was greater from the forepaw cortex to the hindpaw cortex (0.12 mm/ms) than from the hindpaw cortex to the forepaw cortex (0.08 mm/ms). Intriguingly, different activation velocities have been observed within the barrel cortex – with faster activation along the rows compared with the arcs – due to the higher axonal density in the rows [13], [14], [67]. Consequently, the greater activation velocity from forepaw cortex to hindpaw cortex reported here could suggest certain anatomical anisotropy between the forepaw-to-hindpaw and the hindpaw-to-forepaw directions.

On the one hand, the velocities that we obtained here were somewhat higher than those shown by Petersen et al. [13] in the supragranular layers using VSD imaging of the barrel cortex (<0.06 mm/ms). Based on the lower activation velocities that we obtained with low-intensity stimuli, it is indeed expected for the tactile stimuli used by Petersen et al. [13] to evoke slower activation velocities compared with our high-intensity electrical stimuli. On the other hand, the velocity that we obtained in the supragranular layers was lower than the velocity obtained electrophysiologically in the infragranular layers in response to tactile stimulation of the paws [48]. This coincides with the observations of Sakata and Harris [68] regarding the slower expansion velocity in the supragranular layers compared with the infragranular layers. Therefore, we can conclude that the activation velocity of the evoked activity in the supragranular somatosensory cortex depends on both the location and the intensity of stimulation on the body.

The activity expansion that we observed in the supragranular cortical layers in response to somatosensory stimuli may have two possible non-mutually excluding origins: subcortical and cortico-cortical. On the one hand, the subcortical origin is quite important under our experimental conditions: at thalamic level somatosensory stimuli, in addition to the principal receptive field, also activate numerous secondary receptive fields with slightly higher latencies [47], [69]. This would contribute to the rapid expansion of the activity observed at the cortical level. On the other hand, the cortico-cortical origin may have several contributions, as horizontal connections exist both in the input layer 4 [10], [70], in the supragranular layers 2/3 [10], [61], [62] and in the infragranular layers 5/6 [2], [71]. If propagation of cortical activity occurs in one layer, it rapidly extends to the other layers through columnar projections [13], [72], [73].

Whatever the exact mechanism, any contribution to this activity expansion should be a relatively fast event, as the cortical activation reached its maximum extension about 25–30 ms after the stimulus in our experiments. These short latencies clarify that the activations reported here specifically refer to the classical short-latency components of somatosensory responses and not to the long-latency activations that are typically due to the propagation of up-states triggered by the stimuli [51].

### Directionality of Cortical Somatosensory VSD Responses

Stimulation of the forepaw and hindpaw evoked different cortical activation dynamics.

These dynamics had a clear medial directionality in response to forepaw stimulation but were much more uniform in all directions in response to hindpaw stimulation. These observations represent the functional counterpart of the known anatomical projections from the somatosensory to the motor cortex [17], [37]–[41]. Within the sensorimotor cortex, the barrel cortex displays the maximal separation between sensory and motor [36], and somatosensory stimuli first activate the somatosensory cortex and then the motor cortex [17], [40]. Similarly, the forepaw somatosensory cortex is mostly separated from the corresponding region of the motor cortex [35], [36], explaining the large propagation in the medial direction in response to forepaw stimuli in our results. Conversely, the hindpaw somatosensory cortex mostly overlaps with the corresponding region of the motor cortex [35], [36], explaining the uniform propagation in all directions in our results. The overall directionality of cortical VSD responses thus appears to reflect the general preference of somatosensory-evoked activity to propagate from the somatosensory cortex to the corresponding motor cortex.

The different spatio-temporal dynamics of activation between forepaw and hindpaw stimuli could be related to the different functional roles of the paws: the forepaw is more active than the hindpaw in object recognition and knowledge of the outside world [74], [75]. Integrating our observation with previous results in the barrel cortex, it is therefore tempting to speculate that a lower level of overlap between the somatosensory and motor cortex could be necessary for more sophisticated sensorimotor integration. Overall, the activation flow from the somatosensory cortex to the motor cortex could offer the functional substrate for an intriguing “cortical reflex” that could be relevant for sensorimotor control.

### Possible Relevance for Studying Cortical Reorganization

VSD imaging of the paw region of the sensorimotor cortex has been recently used as a tool to investigate cortical reorganization after stroke and after spinal cord injury. On the one hand, stroke in the forelimb somatosensory cortex in mice decreases or eliminates the VSD responses in the affected forelimb area, while altering both the VSD responses in adjacent areas (motor forelimb and hindlimb cortex) [21], [22] and the VSD responses evoked in the hemisphere opposite to the stroke [23]. On the other hand, thoracic spinal cord injury in rats decreases (or eliminates in case of complete lesion) the VSD responses to stimuli delivered to the hindpaw [24], [26], while it increases the activated area to stimuli delivered to the forepaw, leading to a classical expansion of the forelimb cortex into the hindlimb cortex [25]. It is likely that more subtle VSD measures could allow more subtle aspects of cortical reorganization to be uncovered. The novel results reported here – particularly the two ipsilateral foci and the different directionality of cortical propagation between the forepaw cortex and the hindpaw cortex – could, therefore, be helpful to fully understand not only the physiological organization of the rat sensorimotor cortex, but also its reorganization in pathological conditions such as stroke and spinal cord injury.

In conclusion, this work offers a complete spatio-temporal map of the supragranular cortical VSD activation in response to stimulation of the paws, showing important somatotopic differences between contralateral and ipsilateral maps as well as differences in the spatio-temporal activation dynamics in response to forepaw and hindpaw stimuli.

## Materials and Methods

### Surgical Procedures and Dye Staining

All animal experiments described here were performed following the rules of the International Council for Laboratory Animal Science, European Union regulation 2010/63/EU, and were approved by the Ethical Committee for Animal Research of the Hospital Nacional de Parapléjicos (Toledo, Spain). Wistar rats between 280–410 g were anesthetized with urethane (1.0–1.5 g/Kg) applied intraperitoneally. During the experiment, the anesthesia level was regularly monitored by tail-pinch reflex and additional urethane (<20% of original doses) was applied when the reflex appeared. Once the anesthesia had taken complete effect, animals were placed in a stereotaxic frame (SR-6R; Narishige Scientific Instruments, Tokyo, Japan). Lidocaine 2% was applied over body surfaces in contact with the frame and over the incision area. During the experiment, the temperature of animals was kept constant at 36 °C by means of an automatically controlled heating pad. A large craniotomy was performed above the forepaw and hindpaw regions of the primary somatosensory cortex in the left hemisphere (AP = 2:−4; L = 1∶5). Extreme care was taken during craniotomy to maintain the dura intact. In order to decrease the curvature of the cortex due to intracranial pressure, the cisterna magna was opened. Moreover, in order to guarantee that the overall cortical region to be imaged was in the same focal plane, the stereotaxic frame was tilted about 10 degrees. The voltage-sensitive dye RH1691 [11], [14] was dissolved at 2 mg/ml in buffer solution containing (in mM): 126 NaCl, 3.53 KCl, 1.25 NaH_2_PO_4_, 26 NaHCO_3_, 1 MgSO_4_7H_2_O, 10 dextrose, 1000 CaCl_2_). To stain the cortex, the resulting dye solution was topically applied on the craniotomy region and allowed to diffuse in the cortex for 2 h. The craniotomy region was delimited with agar to avoid spilling of the dye solution. After staining, the cortex was washed for 20 min to remove unbound dye. Then, the cortex was covered with 1% agar dissolved in buffer solution and a glass coverslip was placed on top in order to stabilize the cortex [45].

### Imaging VSD Signals

To image the VSD signals, we used light from a 150 W halogen lamp controlled by an electromagnetic shutter (MHAB-150W, MORITEX). The light was band-pass filtered at 632 nm (excitation filter FF01-632/22-25) and reflected toward the cortex by a 650 nm dichroic mirror (FF650-Di01, reflection: 500–640 nm, transmission: 660–825 nm) to excite the dye. The light emitted by the dye from the cortex, after being transmitted without change through the dichroic mirror, was long pass filtered at 665 nm (emission filter RG-665) and finally imaged using a MICAM ULTIMA-L system (BrainVision Inc.). The rationale for this standard VSD recording configuration is that the excitation filter, the dichroic mirror and the emission filter are designed to separate and optimize the excitation fluorescence (smaller wavelength) and the emission fluorescence (larger wavelength) of the RH1691 dye, thereby maximizing the signal-to-noise ratio of the VSD recording. A representative diagram of the experimental setup is showed in Fig. 1.

The camera captures images with 100×100 pixels and the size of the imaged cortical area was 5-mm×5-mm, thus each pixel imaged an area of 50-µm×50-µm. The images were captured with a temporal resolution of 0.5 ms. Non-stimulated sweeps were subtracted to stimulated sweeps in order to minimize the bleaching artifact [45]. In stimulated sweeps, the recording was performed between 250 ms before to 250 ms after the stimulus.

### Electrical Stimulation of the Paws

We used VSD imaging to study the cortical activation evoked by electrical stimulation of the paws. Square-pulse electrical stimuli were generated using a digital stimulator (DS8000) with an ISO-Flex stimulus isolator (A.M.P.I.). Electrical pulses were applied using bipolar needle electrodes located subcutaneously in the wrist of the forepaws and of the hindpaws, one pole on each side of the corresponding paw. The stimulation protocol consisted of a sequence of 30 square-pulses of 2 ms duration and 0.05 Hz frequency. We employed this low frequency of stimulation in order to minimize fototoxicity [12], [14]. We separately applied this stimulation protocol to the four paws, first using low-intensity stimuli (0.6 mA) and then using high-intensity stimuli (6 mA). Low-intensity stimuli were intended to activate only a fraction of the available fibers, mainly low-threshold primary fibers running through the lemniscal pathway, from the dorsal columns to the brainstem [54], [76], [77]. High-intensity stimuli were intended to activate the maximum number of fibers, including high-threshold primary fibers that make synapse in the dorsal horns of the spinal cord, in turn activating the spinothalamic tract [54], [76], [77]. The responses to high-intensity electrical stimuli are more sensitive than the responses to low-intensity electrical or tactile stimuli for investigating cortical reorganization after spinal cord injury [25], [51], [78]–[81]. Note that we could use much higher stimulus strengths compared to previous VSD forepaw/hindpaw studies [24], [25] without saturating the VSD signal, thank to the higher temporal resolution of our acquisition (0.5 ms/frame compared to 5 ms/frame). High-intensity stimuli caused muscle contraction, whereas low-intensity stimuli did not. Muscle contraction corresponds to the m-wave and/or the h-reflex, which in the rat occur between about 1 ms and 10 ms after nerve stimulation [82]. This means that within 10 ms after our high-intensity stimuli the animal received a second somatosensory input due to the muscle contraction. However, it is unlikely that this second input could contribute to our cortical responses due to its much lower intensity and to the strong synaptic depression that occurs for inter-stimulus intervals below 25 ms in the somatosensory system of anesthetized rats [83].

In this study, we used a total of 11 rats: 9 of 11 rats with contralateral stimulation at low intensity, 9 of 11 rats with contralateral stimulation at high intensity and 6 of 11 rats with ipsilateral stimulation.

### Data Analysis

We performed the analysis in a temporal window of 50 ms before the stimulus (background window) and a temporal window of 100 ms after the stimulus (response window) with a temporal resolution of 0.5 ms. To improve the signal-to-noise ratio, we took the average of 30 sweeps and then applied a temporal filter (low pass filter: 200 Hz) and spatial filter (Gaussian low pass filter: #rows = 3, #columns = 3, sigma = 1).

To define the cortical activated region in each temporal instant, we considered the following criteria: the signal value in each pixel must be (1) larger than the mean plus five times the standard deviation of the signal within the background window and (2) at least half the minor of the maximal signal value evoked by different stimuli (in different paws and with different intensities) in any pixel within the response window. The pixels that did not verify the anterior conditions were considered non-activated (value = 0). The conditions for which a pixel was considered activated can be expressed as the following:



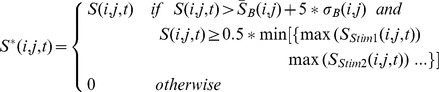


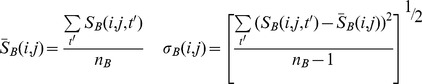
(1)


where 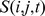 is the initial signal value of the pixel *(i, j)* with *i, j* = 1,2,…,100 at the instant *t* in the response window, and 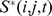 is the signal value assigned to the pixel *(i, j)* at the instant *t*; 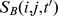
is the signal value of the pixel *(i, j)* at the instant *t'* in the background window;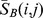 is the mean signal value of the pixel *(i, j)* in the background window; 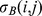
is the standard deviation of the signal in the pixel *(i, j)* in the background window;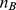 is the number of time points in the background window (100); and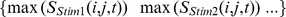 are the maximal signal values for any pixel and any instant in the response windows for the different types of stimuli.

First, we performed an analysis of the VSD response amplitudes and VSD response latencies, which allowed us to compare our VSD results with classical electrophysiological data. Next, making use of the spatio-temporal resolution provided by VSD imaging, we defined several measures to quantify the spatio-temporal dynamics of the cortical activity evoked by the stimuli. The images were exported to Matlab (version 7.1; The Mathworks) for data analysis.

#### Amplitudes and latencies of the cortical somatosensory VSD responses

To study the supragranular cortical VSD responses, we defined the initial response latency, *L*, as the first temporal instant after the stimulus at which there was at least one activated pixel (activated region larger than zero) (Eq. (2)). Among those initially activated pixels, the response amplitude, *Amp*, was defined as the maximal signal value within the response window (Eq. (3)), and the temporal instant in which the signal reached this maximal value was defined as the peak latency, *L_P_* (Eq. (4)).
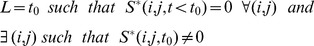
(2)

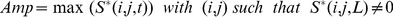
(3)

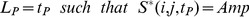
(4)


Initial activation maps were obtained as the first contour (1 ms) from the contour maps of the temporal evolution of the activated region (see *The directionality of cortical somatosensory responses*, below).

#### The extent of cortical somatosensory VSD responses

To quantify the spatial extent of the cortical activation evoked by somatosensory stimuli, we calculated the area of the activated region. For each instant of the response window, the area *A*(*t*) was determined as the number of activated pixels multiplied by the pixel dimension (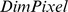 = 0.0025 mm^2^) (Eq. (5)). To study the velocity at which the activation spread over the cortex, or the activation velocity *V*(*t*), we calculated the derivative of the area with respect to the response-window time (Eq. (6)). This activation velocity was measured as mm^2^/ms.
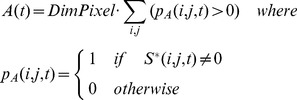
(5)

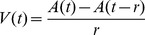
(6)where *r* is the temporal resolution (0.5 ms).

Due to the large extent of the cortex that can respond to a somatosensory stimulus, it is likely that a stimulus delivered to a paw evokes a response in the region of the cortex corresponding to another paw [48]. To investigate the possible cortical overlap between the regions activated by stimulation of forepaw and hindpaw, we determined the maximal cortical overlap, 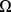, which was calculated as the number of pixels activated by both forepaw and hindpaw stimuli – multiplied by the pixel dimension – in the temporal instant at which the activated area was maximal (Eq. (7)).
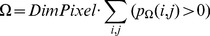
(7)

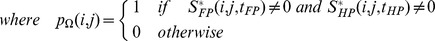



with 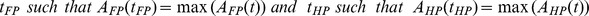 where 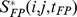 and 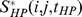 are the signal values assigned to the pixel *(i, j)* when we stimulated the forepaw or hindpaw, respectively.

We further calculated the response amplitude and response latency in the cortical region corresponding to the non-stimulated paw, i.e., the hindpaw cortex when we stimulated the forepaw or the forepaw cortex when we stimulated the hindpaw (i.e. the VSD equivalent of the “non-homologous responses” described in [48]). The response amplitude and response latency were calculated at the same point in which we calculated the amplitude when we stimulated the optimal paw. We also calculated the linear activation velocity from forepaw to hindpaw cortex and the linear activation velocity from hindpaw to forepaw cortex. This velocity was calculated as the distance between forepaw and hindpaw cortex divided by the latency difference between the response in forepaw and hindpaw cortex.

#### The directionality of cortical somatosensory responses

To investigate the possible directionality in the cortical activation dynamics, we first constructed contour maps of the temporal evolution of the activated region. The contours were represented every 1 ms from the instant of initial activation until the instant at which the activated area reached the maximal value. Each contour included all pixels that, at the corresponding time instant, exhibited an intensity greater than a fixed threshold (50% of the maximum intensity among all pixels within the response window). To reduce spatial noise for illustration purposes, regions of less than 10 activated pixels were omitted, and spatial filtering was applied (matlab function ‘imdilate’ with a disk-shape of 2-pixels radius).

To quantitatively study the directionality, we analyzed the spatio-temporal evolution of the center of the activated region (global activation center) from the beginning of the activation until the activated area reached the maximal value. For each temporal instant, the *I* and *J* coordinates of the global activation center were calculated with the same relationship as that for the calculation of a mass center (Eq. (8)).
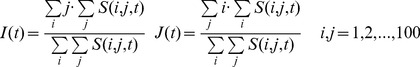
(8)


To study the non-uniformity of the spread direction of the activation, we extended the calculation of the global activation center to the calculation of the activation center in each of the four quadrants of a coordinate system in which the origin was the global activation center in each temporal instant. For each instant *t*, the calculation of the 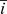 and 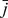 coordinates of the activation center in each quadrant was performed according to the following relationships:



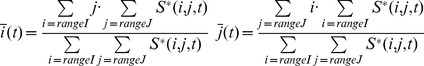
(9)

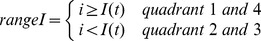
 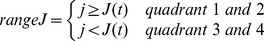 where *I(t), J(t)* are the coordinates of the global activation center and were calculated with Eq. (8).

#### Statistical analyses

To compare the response amplitude and response latency to stimulation of the contralateral forepaw and hindpaw at low and high intensity, we employed a two-way mixed analysis of variance (ANOVA), where the paw was the first factor with two levels of repeated measures (forepaw and hindpaw), and the stimulation intensity was the second factor with two levels of independent measures (low and high intensity). The reason for the independent measures was because not all rats received both low- and high-intensity stimuli. To compare the response amplitude and response latency to stimulation of ipsilateral and contralateral paws, we used a two-way independent measures ANOVA, where the paw was the first factor with two levels (forepaw and hindpaw), and the body side was the second factor with two levels (contralateral and ipsilateral). This comparison was performed only with response to high-intensity stimuli because ipsilateral responses to low-intensity stimuli were not easily detected.

To evaluate the extent of the cortical somatosensory responses, we compared the maximal activated area and the maximal activation velocity of the responses to stimulation of the contralateral forepaw and hindpaw at high and low intensities. This comparison was performed with a two-way mixed ANOVA, where the paw was the first factor with two levels of repeated measures (forepaw and hindpaw), and the stimulation intensity was the second factor with two levels of independent measures (high and low intensity). We compared the overlap between the maximal activated area for stimulation of the contralateral forepaw and hindpaw at high and low intensity using an unpaired *t*-test. The response amplitude and response latency in the cortical region corresponding to the stimulated paw (contralateral forepaw or hindpaw) were compared with the responses in the cortical region corresponding to the non-stimulated paw (contralateral hindpaw or forepaw) using a paired *t*-test. This comparison was performed only with responses to high-intensity stimuli because the responses in the cortical region corresponding to the non-stimulated paw were much less observable at low intensity. The linear activation velocity from the forepaw cortex to the hindpaw cortex was compared with the linear activation velocity from the hindpaw cortex to the forepaw cortex using an unpaired *t*-test.

To study the directionality of the cortical somatosensory responses, we determined if there was movement of the global activation center by comparing the coordinates of the global activation center when the area reached the maximal value with the initial coordinates using a paired *t*-test. This was separately performed for the medial-lateral and anterior-posterior directions for both the contralateral response of forepaw and hindpaw cortex to high-intensity stimulation.

All data were log-transformed for statistical analysis. All results are given as mean ± standard deviation and were considered significant at *p*<0.05.
